# Determinants of Covid-19 vaccine uptake among the elderly aged 58 years and above in Kericho County, Kenya: Institution based cross sectional survey

**DOI:** 10.1371/journal.pgph.0001562

**Published:** 2023-09-12

**Authors:** Calvince Otieno Anino, Immaculate Wandera, Zachary Ondicho Masimba, Collins Kipkosgei Kirui, Carjetine Syallow Makero, Phanice Kerubo Omari, Philip Sanga

**Affiliations:** 1 University of Kabianga, Kericho, Kenya; 2 Ministry of Health, Baringo, Kenya; 3 Maseno University, Maseno, Kenya; Federal University of Mato Grosso do Sul, BRAZIL

## Abstract

Hesitancy to Covid-19 vaccine is a global challenge despite the compelling evidence of the value of vaccine in preventing disease and saving lives. It is suggested that context-specific strategies can enhance acceptability and decrease hesitancy to Covid-19 vaccine. Hence, the study determined uptake and determinants of Covid-19 vaccine following a sustained voluntary vaccination drive by Kenyan government. We conducted institution based cross-sectional survey of 1244 elderly persons aged 58 to 98 years in the months of January, February and March, 2022. A multinomial logistic regression analysis was used to investigate determinants of Covid 19 vaccine uptake. The predictor variables included socioeconomic and demographic characteristics, convenience and ease of access of the vaccine, collective responsibility, complacency and the three dimensions of confidence; trust in safety, trust in decision makers and delivery system. The findings are reported as the adjusted odd ratio (AOR) at 95% confidence interval (CI). Significant level was considered at p <0.05. The results from the multinomial logistic regression analysis indicated that advanced age and presence of chronic disease were associated with increased odds of doubt on Covid 19 vaccine, while long distance from vaccination centers was associated with increased odds of delay in vaccination. Overall, the findings of this study provided valuable insights into the factors influencing vaccine hesitancy among the elderly population in Kenya and will inform the development of targeted interventions to increase vaccine acceptance and uptake in this population.

## Background

Covid-19 vaccine hesitancy, delay in acceptance or refusal of Covid 19 vaccine despite its availability has become a significant global challenge in the effort to control the spread of the pandemic [1]. Despite the overwhelming evidence of the efficacy and safety of vaccines in preventing disease and saving lives [2], some individuals and communities have expressed skepticism or refusal to receive the vaccine. This hesitancy can have serious consequences, as it can lead to decreased vaccine uptake and ultimately contribute to the continued spread of the disease [3,4]. To address this issue, it is important to understand the prevalence and determinants of Covid-19 vaccine hesitancy [5]. A range of factors have been identified as contributing to vaccine hesitancy, including socioeconomic and demographic characteristics, awareness and knowledge of the vaccine, attitudes towards collective responsibility, complacency, and confidence in the vaccine and vaccination process [6,7]. Targeted interventions, such as education campaigns and addressing misinformation, have been shown to be effective in reducing vaccine hesitancy in some populations [8,9]. However, it is essential to recognize that these interventions may not be equally effective in all contexts and that it is necessary to examine the specific factors driving hesitancy in different populations [10,11]. In Kenya, the national government implemented a sustained voluntary vaccination drive as part of its efforts to control the spread of Covid-19 disease with special emphasis on the elderly individuals aged 58 years and above. Despite this, little is known about the prevalence and determinants of vaccine hesitancy in this population. The aim of the study was to fill this gap by conducting a cross-sectional survey of elderly individuals aged 58 years and above in Kenya to investigate the prevalence and determinants of Covid-19 vaccine hesitancy. The results of the study provided valuable information about the specific factors driving vaccine hesitancy in this population and could inform the development of context-specific strategies to increase vaccine acceptability and uptake.

## Methods

### Study design and area

In order to investigate the prevalence and determinants of Covid-19 vaccine hesitancy in Kenya, we conducted an institution-based cross-sectional survey of 1244 elderly individuals aged 58 years and above in three sub-counties of Kericho County in the Southern Rift Valley of Kenya. The participants admitted in the study were aged 58 years or older and were eligible to receive Covid 19 vaccine based on the Kenyan national guidelines for Covid 19 vaccination. These sub-counties were purposively chosen as they have the highest proportion of elderly individuals in the county and together accounted for over 50% of the county’s elderly population. Besides, they had a population range of 9.3% to 11.2% of the elderly persons which was significantly higher than the country’s average of 3.9% as reported by [12].

### Sampling procedure, data collection and ethical consideration

The sampling procedure for this study followed a two-stage approach, involving stratified sampling and systematic random sampling. The aim was to select a representative sample of elderly individuals aged 58 years and older from health facilities offering Covid-19 vaccination in Kericho County. The target population was divided into two strata of all the designated Covid 19 vaccination centers in Kericho County, 12 government health facilities and eight private health facilities. To ensure proportional representation from each stratum, a proportional probability to size (PPS) approach was used in the selection process. The first step involved determining the sample size for each stratum using Fischer’s formula for estimating proportions, with a desired level of precision set at ±5%, a confidence level of 95%, a margin of error of 3%, and a prevalence rate of 50%. The sample size calculation was adjusted proportionally to the size of each stratum, 740 for government health facilities and 504 for private health facilities. Next, the total sample size for each stratum was divided by the number of facilities within that stratum to determine the number of respondents to be selected from each facility. This ensured that larger facilities had a higher probability of being selected. For participant selection within each facility, a systematic random sampling method was employed. A list of eligible elderly individuals was obtained from the health records, and unique identification numbers were assigned to each individual. Then, a systematic sampling interval was determined by dividing the total number of eligible individuals by the desired sample size. Starting at a randomly computer-generated number within the sampling interval, every nth individual on the list was selected until the required number of respondents was reached for each facility.

The questionnaires were administered in private rooms by trained interviewers, who were graduate public health students on their 10th month of internship and had knowledge of the 5Cs of vaccine hesitancy (confidence, complacency, convenience, and collective responsibility). The interviews were conducted in a confidential manner, with coded lists used to assign unique codes to each respondent in order to maintain their anonymity. Tablet computers with the open data kit collect application were used by the interviewers to collect the data, which was then submitted to a central server at the end of each day. The study was approved by the University of Kabianga Institutional Research Ethics Committee and written informed consent was obtained from each respondent.

### Study variables

#### Demographic characteristics

In order to gather demographic information, we collected data on participants’ age, gender, marital status, level of education, and occupation. Age was recorded as a continuous variable but was later transformed into a categorical variable for analysis, with the categories being under 70 years, 70 to 80 years, and above 80 years. We also asked participants about their primary occupation and whether they had any history of chronic disease or had taken any vaccine in the last five years. In addition, we recorded whether participants were healthcare workers and their area of residence. All of this information was collected using yes or no responses.

#### Covid 19 vaccine uptake categories

The primary focus of this study was to assess the determinants of Covid-19 vaccine uptake. To do this, we first asked participants whether they were aware of the Covid-19 vaccine. We then measured vaccine uptake by asking participants if they had received any of the Covid-19 vaccines. We used a single item measure with four possible responses as described by [13]: (1) accepting the vaccine without doubt for reasons other than allergies or illness, (2) accepting the vaccine with doubt for reasons other than allergies or illness, (3) refusing the vaccine for reasons other than allergies or illness, or (4) delaying the vaccine for reasons other than allergies or illness. Based on their responses, participants were classified into one of four categories; refusers, delayers, acceptors with doubt, or no vaccine hesitancy. We used the classification criteria defined by [13] in order to classify vaccine acceptance and hesitancy.

#### Determinants of Covid 19 vaccine uptake

To assess the determinants of Covid-19 vaccine uptake, we used a modified version of the 5C model of the psychological antecedents to vaccination [2,13]. We used a 5-point scale as described by [13] instead of 7-point scale. This model suggests that complacency, constraints, confidence, collective responsibility, and calculation are important predictors of vaccine hesitancy. We assessed confidence in three dimensions as described by [13]: trust in the safety and effectiveness of the Covid-19 vaccines, trust in the government officials who make decisions about the Covid-19 vaccines, and trust in the delivery of the Covid-19 vaccination services with regards to the competency and reliability of the healthcare workers. We used a 10-item scale with a 5-point hedonic response scale to measure the extent to which participants agreed with these dimensions of confidence. The variables measured in the dimension of trust in vaccine safety included concerns about safety, unknown side effects, long-term effects, harmful substances in the vaccine, and the short development time of the vaccine. We also assessed trust in the safety and effectiveness of the vaccine with regard to religion compatibility and used a scoring system with responses ranging from 1 (strongly disagree) to 5 (strongly agree). Trust in the delivery of the Covid-19 vaccination services was measured using a similar scoring system, with responses ranging from 1 (strong distrust) to 5 (strong trust) for vaccine manufacturers, professional institutions, and healthcare providers. We also used a similar scoring system to assess trust in decision-makers, including government officials, politicians, and church leaders. For the purpose of analysis, responses to each of the 10 items were further classified into two categories: agree (including responses of "strongly agree" and "agree" for trust in vaccines, and "strong trust" and "trust" for the Covid-19 vaccination delivery system and decision-makers) and disagree (including responses of "strongly disagree" and "disagree" for trust in vaccines, and "strong distrust" and "distrust" for the Covid-19 vaccination delivery system and decision-makers).

We also measured collective responsibility using a 3-point scale, with responses of "always," "sometimes," and "never" to questions about mask-wearing, physical distancing, and hand hygiene in public and at home. To assess convenience the authors used both ‘Yes or No’ responses and a Likert scale with responses ranging from 1 (strongly disagree) to 5 (strongly agree). The later was used to assess ‘do you feel you have enough time to receive Covid 19 vaccine’ and the former was used in the following two questions. ‘Is the distance to the vaccination centers a barrier for you in getting Covid 19 vaccine?’ and ‘Did you encounter difficulties in securing an appointment for the COVID-19 vaccine?’. Complacency was measured using a Likert scale with responses ranging from 1 (Not at all) to 5 (very high) for the first question, 1 (Strongly disagree) to 5 (Strongly agree) for the second question and 1 (Not important at all) to 5 (Extremely important) for the third question. The questions asked were. ‘To what extent do you believe that you are at risk of contracting COVID-19?’, ‘do you agree with the statement ‘I am generally against vaccines?”, and lastly ‘COVID-19 vaccine is not important to my health and the health of others?’.

### Data analysis

To analyze the data collected from this study, we used multinomial logistic regression analysis to investigate the prevalence and determinants of Covid-19 vaccine hesitancy in the elderly population in Kenya. The predictor variables, or potential determinants of vaccine hesitancy, were socioeconomic and demographic characteristics, awareness of the Covid-19 vaccine, attitudes towards collective responsibility, complacency, and the three dimensions of confidence. The dependent variable was the vaccine hesitancy status of each participant, as classified into one of four categories: refusers, delayers, acceptors with doubt, or no vaccine hesitancy.

We presented the findings as adjusted odds ratios (AOR) at a 95% confidence interval (CI). A p-value of less than 0.05 was considered statistically significant. We also conducted subgroup analyses to examine any potential differences in vaccine hesitancy among different demographic subgroups. Additionally, we used descriptive statistics to summarize the sociodemographic characteristics of the study sample and to provide an overview of the prevalence of vaccine hesitancy among the elderly population in Kenya. We also used cross-tabulations and chi-square tests to explore any potential associations between vaccine hesitancy and the various predictor variables included in the analysis.

## Results

### Socio-demographic characteristics and vaccine uptake

The results of this study showed that, among the elderly population in Kenya, a significant proportion expressed hesitancy towards the Covid-19 vaccine (Table 1). Of the respondents, 81.5% were aware of the vaccine, but only 27.4% were non hesitant, while 14.5% accepted it with doubt, 37.1% were delayers, and 21% were refusers.

**Table 1:** Socio-demographic characteristics of the respondents and Covid 19 vaccine uptake level.

Variables	n (%)	Covid 19 vaccine uptake				*P*-value
		No hesitancy	Acceptors with doubts	Delayers	Refusers	
	1244	341 (27.4)	180 (14.5%)	462 (37.1%)	261 (21%)	
Gender						0.63
Male	692 (55.6%)	164 (48.1%)	106 (58.9%)	297 (64.3%)	125 (47.9%)	
Female	552 (44.4%)	177 (51.9%)	74 (41.1%)	165 (35.7%)	136 (52.1%)	
Age category						0.01*
<70 years	913 (73.4%)	236 (69.2%)	139 (77.2%)	354 (76.6%)	184 (70.5%)	
70 to 80 years	281 (22.6%)	91 (26.7%)	34 (18.9%)	97 (21%)	59 (22.6%)	
>80 years	50 (4%)	14 (4.1%)	7 (3.9%)	11 (2.4%)	18 (6.9%)	
Marital status						<0.01**
Single	30 (2.4%)	6 (1.8%)	13 (7.2%)	6 (1.3%)	5 (1.9%)	
Married	1194 (96%)	333 (97.6%)	159 (88.3%)	448 (97%)	254 (97.3%)	
Divorced	20 (1.6%)	2 (0.6%)	8 (4.5%)	8 (1.7%)	2 (0.8%)	
Level of education						0.02*
Primary	478 (38.4%)	158 (46.3%)	13 (7.2%)	196 (42.4%)	111 (42.5%)	
Secondary	295 (23.7%)	44 (12.9%)	116 (64.4%)	88 (19%)	47 (18%)	
Post-secondary	214 (17.2%)	123 (36.1%)	43 (23.9%)	20 (4.3%)	28 (10.7%)	
None	257 (20.7%)	16 (4.7%)	8 (4.4%)	158 (34.2%)	75 (28.7%)	
Primary occupation						0.03*
Farming	766 (61.6%)	139 (40.8%)	94 (52.2%)	298 (64.5%)	235 (90%)	
Business	175 (14.1%)	43 (12.6%)	61 (38.9%)	65 (14.1%)	6 (2.3%)	
Informal	303 (24.4%)	159 (46.6%)	25 (13.9%)	99 (21.4%)	20 (7.7%)	
Residence						0.88
Kericho County	953 (76.6%)	259 (76%)	121 (67.2%)	389 (84.2%)	184 (70.5%)	
Other counties	291 (23.4%)	82 (24%)	59 (32.8%)	73 (15.8%)	77 (29.5%)	
Healthcare worker						0.61
Yes	58 (4.7%)	43 (12.6%)	9 (5%)	4 (0.9%)	2 (0.8%)	
No	1186 (95.3%)	298 (87.4%)	171 (95%)	458 (99.1%)	259 (99.2%)	
Chronic disease						<0.01**
Yes	682 (54.8%)	218 (63.9%)	24 (13.3%)	302 (65.4%)	138 (52.9%)	
No	562 (45.2%)	123 (36.1%)	156 (86.7%)	160 (34.6%)	123 (47.1%)	
Awareness						
Yes	1014 (81.5%)	341 (100%)	180 (100%)	317 (68.6%)	176 (67.4%)	
No	230 (18.5%)			145 (31.4%)	85 (32.6%)	

* Indicates statistical significance (p < 0.05).

In terms of sociodemographic characteristics, all variables, except for gender and county of residence, were significantly associated with vaccine uptake. Those who were married or aged below 70 years were more likely to be classified as acceptors with doubts, delayers, or have an intention to refuse the vaccine, compared to their respective cohort categories. Additionally, those with secondary or post-secondary education were more likely to be classified as acceptors with doubts, and a significant proportion of farmers had an intention to refuse the vaccine.

### Association of confidence dimensions, collective responsibility, convenience and complacency with Covid 19 uptake

Tables 2 and 3 show the association of confidence dimensions, collective responsibility, and complacency with Covid-19 vaccine uptake. All three dimensions of confidence were significantly associated with vaccine uptake. Trust in vaccine safety was significantly associated with no hesitancy (87.7%), while trust in the delivery system was significantly associated with no hesitancy (94.7%) and acceptance with doubts (63.9%). Specific trust parameters, such as trust in the ability of the vaccine to protect, were also significantly associated with no hesitancy and acceptance with doubts. However, concerns about long-term effects and unknown side effects were highly associated with acceptance with doubts, delay, and refusal.

**Table 2:** Association between confidence dimension and Covid 19 uptake.

Variables	Covid 19 vaccine uptake				*P*-value
	No hesitancy 341 (27.4%)	Acceptors with doubts 180 (14.5%)	Delayers 462 (37.1%)	Refusers 261 (21%)	
**Safety**					
Vaccine is effective	93 (27.3%)	146 (81.1%)	238 (51.5%)	71 (27.2%)	0.03*
Unknown side effect	124 (36.4%)	150 (83.3%)	420 (90.9%)	251 (96.2%)	0.01*
Long term effects	143 (41.9%)	134 (74.4%)	401 (86.8%)	247 (94.6%)	0.01*
Harmful substance	57 (16.7%)	37 (20.6%)	86 (18.6%)	54 (20.7%)	0.25
Too short time for development and testing	32 (9.4%)	66 (36.7%)	194 (42%)	196 (75.1%)	0.02*
Vaccine is compatible with personal beliefs	96 (28.2%)	112 (62.2%)	237 (51.3%)	74 (28.4%)	3.41
Vaccine is compatible with natural remedies	85 (25%)	107 (59.4%)	221 (47.8%)	65 (24.9%)	0.72
Covid 19 vaccine completely protect people who take it	270 (79.2%)	126 (70%)	193 (41.7%)	60 (23%)	0.01*
I/someone I know had a bad experience with previous vaccine	197 (57.7%)	12 (6.7%)	22 (4.8%)	24 (9.2%)	0.07
I/someone I know had bad experience with Covid 19 vaccine	13 (4%)	104 (57.8%)	237 (51.3%)	151 (57.9%)	0.02*
Trust vaccine is safe for use					<0.01**
Agree	299 (87.7%)	58 (32.2%)	124 (26.8%)	20 (7.7%)	
Disagree	42 (12.3%)	122 (67.8%)	338 (73.2%)	241 (92.3%)	
**Decision makers**					
Government officers	328 (96.2%)	151 (83.9%)	375 (81.2%)	112 (42.9%)	0.01*
Church leaders	337 (98.8%)	163 (90.5%)	413 (89.4%)	194 (74.3%)	0.01*
Politicians	114 (33.4%)	79 (43.9%)	262 (56.7%)	223 (85.4%)	<0.01**
Trust in decision makers					0.04*
Agree	329 (96.5%)	170 (94.5%)	431 (93.3%)	341 (92.3%)	
Disagree	12 (3.5%)	11 (6.5%)	32 (6.7%)	20 (7.7%)	
**Delivery system**					
Healthcare workers	309 (90.6%)	160 (88.9%)	388 (84%)	123 (47.1%)	0.01*
Hospital and other professional institutions	314 (92.1%)	167 (92.8%)	433 (93.7%)	189 (72.4%)	0.03*
Vaccine manufacture and companies	292 (85.6%)	126 (70%)	289 (62.6%)	135 (51.7%)	0.01*
Trust in delivery system					0.01*
Agree	323 (94.7%)	115 (63.9%)	207 (44.8%)	90 (34.5%)	
Disagree	18 (5.3%)	65 (36.1%)	255 (55.2%)	171 (65.5%)	

* Indicates statistical significance (p < 0.05).

**Table 3:** Association of collective responsibility, convenience and complacency with Covid 19 vaccine uptake.

Variables	Covid 19 vaccine uptake				*P*-value
	No hesitancy 341 (27.4%)	Acceptors with doubts 180 (14.5%)	Delayers 462 (37.1%)	Refusers 261 (21%)	
**Collective responsibilities**					
I wear face mask	287 (84.2%)	118 (65.7%)	270 (58.4%)	144 (55.2%)	<0.01*
I keep physical distance	135 (39.6%)	45 (24.9%)	102 (22.1%)	9 (3.5%)	0.63
I wash hands in public place	162 (47.5%)	73 (40.6%)	144 (31.2%)	45 (17.2%)	0.04*
I wash hands at home	117 (34.3%)	59 (32.8%)	106 (22.9%)	36 (13.8%)	0.06
Take collective responsibility					0.02*
Agree	284 (83.3%)	73 (40.6%)	144 (31.2%)	46 (17.6%)	
Disagree	57 (16.7%)	107 (59.4%)	318 (68.8%)	215 (82.4%)	
**Convenience**					
I don’t have time	-	17 (9.4%)	53 (11.5%)	11 (4.2%)	1.33
Distance is far	-	51 (28.3%)	191 (41.3%)	87 (33.3%)	0.04*
Poor quality of healthcare in terms of how they handle patient appointments	-	3 (1.7%)	11 (2.4%)	-	0.87
**Complacency**					
I don’t think I will be infected	36 (10.6%)	41 (22.8%)	97 (21%)	102 (39.1%)	0.03*
I am against vaccines in general	7 (2.1%)	34 (18.9%)	462 (100%)	261 (100%)	0.56
Vaccine is not important	114 (33.4%)	115 (63.6%)	195 (42.3%)	87 (33.3%)	0.02*
Complacent to receive vaccine					0.04*
Agree	124 (36.4%)	137 (76.1%)	395 (85.5%)	234 (89.7%)	
Disagree	217 (63.6%)	43 (23.9%)	67 (14.5%)	27 (10.4%)	

* Indicates statistical significance (p < 0.05).

Collective responsibility was highly associated with no hesitancy (83.3%), with wearing a face mask and washing hands in public places being the only significant parameters. Acceptance with doubts and intentions to delay or refuse the vaccine were negatively associated with the distance from one’s home to the Covid-19 vaccination center.

A significant proportion of those who expressed some degree of complacency towards the Covid-19 vaccine were more likely to accept the vaccine with doubts (76.1%), delay (85.5%), or refuse (89.7%) it. This was particularly true among those who thought they would not get infected and those who perceived that Covid 19 vaccine was not important.

### Determinants of Covid 19 vaccine hesitancy

Multinomial logistic regression was used to analyze the determinants of Covid-19 vaccine uptake, with no hesitancy as the reference category (Table 4). The results showed that respondents aged below 70 years were more likely to accept the vaccine with doubts (AOR = 16.4; 95% CI = 15.92–20.76), while those aged 80 years were less likely to accept the vaccine with doubts (AOR = 0.33; 95% CI = 0.06–0.49) compared to their older counterparts. Level of education was significantly associated with Covid-19 vaccine uptake, with respondents with secondary education having higher odds of accepting the vaccine with doubts (AOR = 2.99; 95% CI = 2.07–4.18) or refusing it altogether (AOR = 4.10; 95% CI = 3.72–6.45) compared to the reference category. Post-secondary education was significantly associated with higher odds of accepting the vaccine with doubts (AOR = 2.11; 95% CI = 1.76–2.80), delaying (AOR = 3.13; 95% CI = 1.91–4.15), or refusing (AOR = 3.02; 95% CI = 2.47–4.38) it. Respondents with chronic diseases had higher odds (AOR = 2.12; 95% CI = 1.53–3.37) of accepting the Covid-19 vaccine compared to the no hesitancy reference group. Trust in decision makers was significantly associated with a higher likelihood of refusing the Covid-19 vaccine (AOR = 2.59; 95% CI = 2.31–3.04). Collective responsibility was negatively associated with the likelihood of accepting the vaccine with doubts (AOR = 4.12; 95% CI = 3.76–4.91), delaying (AOR = 0.05; 95% CI = 0.04–0.06), or refusing (AOR = 0.30; 95% CI = 0.25–0.40) it. Elderly respondents who had to travel long distances were more likely to delay their first Covid-19 vaccination dose (AOR = 2.64; 95% CI = 1.62–4.71). Complacency was also significantly associated with the intention to delay (AOR = 1.83; 95% CI = 1.30–2.31) or refuse (AOR = 3.40; 95% CI = 2.98–4.30) the Covid-19 vaccine. Marital status, primary occupation, and trust in vaccine safety and delivery system were not significantly associated with the uptake of the Covid-19 vaccine.

**Table 4:** Determinants of Covid 19 vaccine uptake by multinomial logistic regression (no hesitancy as reference category).

Variables	Acceptors with doubts	Delayers	Refusers
Age category			
<70 years	16.4 (15.92, 20.76) *	1.97 (1.22, 2.56)	3.06 (2.23, 4.19)
70 to 80 years	3.52 (2.86, 6.04)	0.72 (0.09, 1.62)	3.50 (2.41, 5.56)
>80 years	0.33 (0.06-0.49) **	1.63 (0.44, 2.51)	1.72 (0.89, 4.38)
Marital status			
Single	3.7 (2.88, 6.31)	3.89 (2.23, 5.19)	1.33 (0.31, 2.07)
Married	0.15 (0.07, 0.34)	3.98 (1.37, 6. 55)	2.0 (0.43, 3.61)
Divorced	1.89 (0.61, 5.85)	1.82 (0.36, 7.13)	3.71 (0.41, 5.56)
Level of education			
Primary	0.32 (0.76, 1.32)	0.53 (0.15, 1.92)	0.33 (0.09, 1.24)
Secondary	2.99 (2.07, 4.18) *	0.36 (0.08, 1.63)	4.10 (3.72, 6.45) *
Post-secondary	2.11 (2.01, 2.80) *	3.13 (1.91, 4.15) *	3.02 (2.47, 4.38) *
None	1.04 (0.23, 5.33)	0.72 (0.41, 1.01)	0.48 (0.27, 1.53)
Primary occupation			
Farming	0.88 (0.18, 4.42)	0.08 (0.01, 2.42)	0.82 (0.15, 2.41)
Business	0.91 (0.14, 6.04)	0.53 (0.11, 2.55)	0.34 (0.03, 3.69)
Informal	1.34 (0.89, 5.76)	0.57 (0.09, 3.73)	1.59 (0.36, 5.10)
Chronic disease	2.12 (1.53-3.37) **	0.57 (0.19, 1.71)	2.48 (0.61, 4.06)
Trust vaccine is safe for use	1.06 (0.29, 3.86)	3.77 (3.35, 4.12)	0.35 (0.31, 0.38)
Trust in decision makers	1.89 (1.62, 2.11)	3.47 (2.83, 3.65)	2.59 (2.31, 3.04) **
Trust in delivery system	3.57 (1.63, 7.32)	2.00 (1.71, 2.38)	2.83 (2.47, 2.97)
Take collective responsibility	4.12 (3.76, 4.91) *	0.05 (0.04, 0.06) *	0.30 (0.25, 0.40) *
Distance is far	2.64 (1.62, 4.71) *	1.38 (0.72, 3.69)	4.05 (2.66, 5.33)
Complacent to receive vaccine	0.93 (0.56, 2.23)	1.83 (1.30, 2.31) *	3.40 (2.98, 4.30) **

* Indicates statistical significance (p < 0.05).

** Indicates statistical significance (p < 0.01).

## Discussion

### Socio-demographic characteristics

In this study, we found that age, education, and primary occupation were key determinants of vaccine acceptance and hesitancy among the elderly population in Kenya. According to [12] there are more females (55.8%) than males (44.2%) in the country. However, in our study there were more males than females. This variation could be due to differences in health seeking behavior among the study population. Previous studies showed that younger age, lower education, and working in informal sectors were often associated with vaccine hesitancy [13]. However, in our study, we found that being below the age of 70 was a predictor of vaccine acceptance with doubts, but not resistance or delay. In contrast, being above the age of 80 was associated with a decrease in the odds of hesitancy. This may be due to the fact that the elderly population, who are at the greatest risk of adverse Covid-19 disease outcomes [14,15], were intentionally targeted for vaccination.

We also found that education was significantly associated with vaccine uptake, with those with secondary or post-secondary education more likely to be classified as acceptors with doubts. This may be because the most educated in a population group are often the major users of health services and are more able to understand health promotional messages [16,17]. However, in our study, the most educated were more likely to hesitate taking the vaccine. This was probably due to the type of information they assessed, as well as their high trust in the opinions of family and friends and political leaders for decision-making. Besides, considering the cultural context in which our study was conducted, the prevalence of traditional medicine use in the area could have implications for vaccine acceptance [18]. In settings where traditional medicine plays a significant role, it is possible that individuals may have concerns or beliefs related to vaccines that are influenced by traditional healing practices [19]. This could probably explain the higher hesitancy among the respondents when compared with those from other regions [20].

### Confidence in Covid 19 vaccine

Several studies conducted in western populations have reported an association between a short development time for a vaccine and vaccine uptake [21,22]. However, the present study did not find such a relationship. Instead, fear that the vaccine contained harmful substances was significantly associated with distrust in the vaccine’s safety [23]. This finding confirms an earlier report by [24], which showed an increased odds of vaccine refusal among the African population due to misconceptions about the vaccine’s contents. While there are misconceptions about the content of the vaccine [25], government officials have consistently educated the public about the development process for the Covid-19 vaccine [26]. Therefore, distrust in the vaccine’s safety is a cause for concern given the extensive sensitization efforts by both the county and national governments and the high number of respondents who had a lot of information about the Covid-19 vaccine.

The source and type of information provided are important factors in facilitating behavior change and acceptance of intervention processes and outcomes [27]. Similarly, studies by [28] have reported increased utilization of vaccination services among the most informed population groups, leading to low vaccine hesitancy. They reported high acceptance of measles and tetanus vaccines among population groups who received information from public health officers, nurses, and community health workers, with local vernacular radio stations as the medium of delivery. Our findings are consistent with these reports, but it is important to consider the roles played by other factors in the 5Cs model in enabling vaccine acceptance [26].

Severe Covid-19 disease and worse outcomes have been associated with comorbidities, particularly among cases with Delta and Kappa coronavirus strains [29]. A high case fatality rate (40%) has been reported among Covid-19 patients with diabetes and cardiovascular diseases [26]. In the present study, a significant proportion of respondents with comorbidities were diabetic and were more likely to hesitate taking the vaccine. If previous reports are to be believed, this hesitation may be due to misconceptions and negative information about severe reactions to the vaccine and the harmful substances they contain, leading to fear of worse health outcomes [28]. While there are limited studies in this area, it is not clear that increased vaccine uptake would lead to higher fatality rates or adverse health outcomes. However, if the rare cases of health outcomes after receiving the first or second jab of vaccines are considered, there is a potential for a reaction to the vaccine, regardless of the type of vaccine or manufacturer [21]. Further studies in this emerging area are recommended, as it was outside the scope of our study.

### Other 5Cs of vaccine hesitancy

The respondents perceived that they would not be infected with Covid 19 disease and they could not relate with the importance of Covid 19 vaccine. Complacency to vaccine can be prevented through targeted health promotion, such as by providing education and awareness campaigns [30,31]. This leads to positive perception about the vaccine and raises awareness on the importance of the vaccine to the population [32,33]. One key reason in our study which contributed to complacency and is previously reported was the perception that one is not likely to be infected with the disease and general feeling of having enough information to keep one safe [34]. Additionally, past studies showed negative association between complacency and collective responsibilities [30]. Threefold likelihood of complacency was observed among the respondents that had recommendable scores for collective responsibilities [35]. The findings for the present work were in tandem with these earlier reports [36–39]. Additionally, study by [40] observed that risk communications targeting both complacency and collective responsibility is likely to boost uptake of Covid 19 vaccine. However, in order to make a conclusive decision, more work need to be done in this area.

In developed countries not having time was the main reported reason for lack of convenience [41]. Similar studies in developing countries have reported two major reasons for lack of convenience in Covid 19 vaccine to be long distance from the vaccination centers and homesteads and lack of knowledge about the vaccinations [42,43]. Our study agreed with these findings since long distance was associated with nearly threefold likelihood of hesitancy. Indeed, earlier reports showed low child immunization rates among caregivers living several miles away from health facilities and among the elderly [44–46]. Therefore, it is evident from our findings and earlier studies that lack of convenience and lack of knowledge about the vaccine are major reasons for hesitancy.

### Implications of results

The findings of the study have important implications for public health practice and when implemented could effectively address vaccine hesitancy and promote vaccine acceptance.

1. To start with, there is need for targeted health promotion efforts. This could be done by educating the population about vaccine safety and raising their awareness about the development process, and benefits. Additionally, there is need for tailored campaigns taking into account cultural and regional contexts and could be led by the opinion leaders such as healthcare professionals, community health workers, and local media channels for best results.
2. It is important to address distrust in the vaccine’s safety. As a matter of priority, concerns and misconceptions regarding the vaccine’s contents should be addressed since they were among the top determinants of hesitancy in our study. This can be done through transparent communication and giving information about the rigorous process it takes to develop vaccines and the absence of harmful substances. These are essentials in building trust among hesitant individuals.
3. There is need for tailored intervention among the elderly with comorbidities since they were more likely to exhibit vaccine hesitancy. The interventions could address concerns related to vaccine safety and potential adverse reactions. This could be done by providing accurate information about the vaccine’s benefits in preventing severe COVID-19 outcomes among those with comorbidities.
4. Convenience and accessibility are key to vaccine acceptance and therefore should be top priority. They could be achieved through deliberate efforts to facilitate easy access to vaccination centers, especially among those living in remote areas. Accessibility could be enhanced by establishing additional vaccination centers, providing transportation services, and organizing mobile vaccination units.

## Conclusion

In conclusion, our study found that age and education were the major significant socio-demographic factors in vaccine acceptance and hesitancy among the elderly population in Kenya. Fear that the vaccine contained harmful substances was significantly associated with distrust in the vaccine’s safety, while lack of convenience was associated with hesitancy. In addition, perception that one is not likely to be infected with Covid 19 and that vaccine is not important were associated with complacency.
